# Physiological and Behavioural Responses to Noxious Stimuli in the Atlantic Cod (*Gadus morhua*)

**DOI:** 10.1371/journal.pone.0100150

**Published:** 2014-06-17

**Authors:** Jared R. Eckroth, Øyvind Aas-Hansen, Lynne U. Sneddon, Helena Bichão, Kjell B. Døving

**Affiliations:** 1 Department of Molecular Biosciences, University of Oslo, Oslo, Norway; 2 Norwegian Institute of Food, Fisheries and Aquaculture Research, Nofima, Tromsø, Norway; 3 Institute of Integrative Biology, University of Liverpool, Liverpool, United Kingdom; University of Windsor, Canada

## Abstract

In the present study, our aim was to compare physiological and behavioural responses to different noxious stimuli to those of a standardized innocuous stimulus, to possibly identify aversive responses indicative of injury detection in a commercially important marine teleost fish, the Atlantic cod. Individual fish were administered with a noxious stimulus to the lip under short-term general anaesthesia (MS-222). The noxious treatments included injection of 0.1% or 2% acetic acid, 0.005% or 0.1% capsaicin, or piercing the lip with a commercial fishing hook. Counts of opercular beat rate (OBR) at 10, 30, 60, 90 and 120 min and observations of behaviour at 30 and 90 min post-treatment were compared with pre-treatment values and with control fish injected with physiological saline, an innocuous stimulus. Circulatory levels of physiological stress indicators were determined in all fish at 120 minutes post-treatment. All treatments evoked temporarily increased OBR that returned to pre-treatment levels at 60 minutes (saline, 0.005% capsaicin, hook), 90 minutes (0.1% acetic acid, 0.1% capsaicin), or 120 minutes (2% acetic acid), but with no significant differences from the control group at any time point. Fish treated with 0.1% and 2% acetic acid and 0.1% capsaicin displayed increased hovering close to the bottom of the aquaria and fish given 2% acetic acid and 0.1% capsaicin also displayed a reduced use of shelter. The only effect seen in hooked fish was brief episodes of lateral head shaking which were not seen pre-treatment or in the other groups, possibly reflecting a resiliency to tissue damage in the mouth area related to the tough nature of the Atlantic cod diet. There were no differences between groups in circulatory stress indicators two hours after treatment. This study provides novel data on behavioural indicators that could be used to assess potentially aversive events in Atlantic cod.

## Introduction

The ability of animals to detect stimuli that can cause harm to their tissues is a universal feature termed nociception [1]. This sensory ability encompasses the neural processing of noxious stimuli and may include the induction of both physiological and behavioural responses but with the sensation of pain not necessarily being implied [2]–[4]. In contrast, pain is regarded as an aversive sensory and emotional experience, representing awareness by the animal of actual or potential tissue damage [2], [5]. In fishes, it has been argued that the neural processing of noxious stimuli may involve peripheral and central nociception but does not allow for pain perception, and that behavioural responses to noxious stimuli may represent an activation of nocifensive motor programs which does not involve conscious awareness [6], [7]. In contrast, other studies have demonstrated nociception and suggested the existence of affective states and potentially the ability for pain perception in teleost fishes (reviewed by e.g. [8]–[11]). Studies on the rainbow trout (*Oncorhynchus mykiss*), for example, have demonstrated A-delta and C-fibre nociceptors [12]–[15] and shown that subcutaneous injections of acetic acid or bee venom to the lips caused enhanced opercular beat rate (OBR) and prolonged time to resume feeding [15]. The trout showed rocking body movements or rubbing its lips against the wall of the tank or in the gravel that were ameliorated by the use of an analgesic [14], [16]. Adverse changes in behaviour and physiology have also been observed in studies of goldfish (*Carassius aurata*) and Nile tilapia (*Oreochromis niloticus*) [17]–[19]. Furthermore, in common carp (*Cyprinus carpio*) and zebrafish (*Danio rerio*), some behavioural and physiological responses to noxious stimuli were similar to those seen in the rainbow trout, whereas other behaviours were found to differ between these species [20].

In the present study, our aim was to evaluate whether some well-characterized noxious stimuli elicit physiological and behavioural responses differing from those induced by the administration of a standardized innocuous stimulus, thus possibly identifying aversive responses associated with injury detection. Specifically, we have investigated if the administration of acetic acid, capsaicin or a commercial fishing hook to the lip of the fish induced physiological and/or behavioral responses differing from the responses seen when administering physiological saline, a standardized innocuous stimulus [15]. Atlantic cod (*Gadus morhua*) was chosen as a marine teleost model due to the large body of general knowledge on the biology of this species and the need for increased knowledge on the fish welfare of this species due to its commercial importance in North Atlantic fisheries and aquaculture. To our knowledge, this is the first study to specifically investigate physiological and behavioral effects of noxious chemicals in a marine teleost, including the first observation of possible nociception with capsaicin in fish, a frequently used stimulus in mammalian pain research [21]. In addition, we are not aware of previous studies where possible effects of the presence of a commercial fishing hook has been investigated and compared to the effects of more commonly used tests in nociception and pain research. In order to facilitate inter-specific comparisons, we employed methods and experimental design evaluated in previous fish studies [12], [15], [16], [22]–[24], including the standardized use of temporary acute anaesthesia during stimulus administration [25]. We have expanded on most previous studies of nociception in fish by including measurements of circulatory levels of commonly used primary and secondary stress indicators in fish, to identify possible differences between treatments as nociception and pain is inherently stressful [26]–[28].

## Materials and Methods

### Ethics Statement

All experiments were conducted in accordance with the Norwegian Fish Welfare and Laboratory Animals legislation [29], [30], which adheres to the European Convention for the Protection of Vertebrates used for Experimentation and other Scientific Purposes [31]. The protocol was approved by the Committee on the Ethics of Animal Experiments of the University of Oslo (Permit Number: 3052).

### Animals and Rearing Conditions

Juvenile Atlantic cod (275±66 g, 30.7±2.4 cm, n = 42) were obtained from Tromsø Aquaculture Research Station, Tromsø, Norway. The experiments were performed at the University of Oslo, Norway, in the period 16. February –30. March 2011. Before experiments, cod were distributed in two stock tanks (217×90×93 cm) with a constant flow (720 L·h**^−^** ^1^) of filtered aerated saltwater (Hydrotech, Filter type Hdt 501-1p; 3.2% salinity, Marinium Reef Sea Salt), and were fed an appropriate diet (3% of the body mass, 5 mm pellets – Aglo Norse Coldwater ekstra, Eximo A/S). The fish were kept at a water temperature of 9±1°C and a 12 h:12 h light:dark cycle. Each cod was given a minimum of two weeks to acclimate in the stock tanks before they were used in experiments.

### Experimental Protocol

Cod were caught at random and placed individually in one of ten rectangular glass aquaria (approximately 150 L; 110×35×40 cm) with a flow (60 L·h**^−^** ^1^) of aerated seawater (water temperature of 9±1°C and a 12 h:12 h light:dark cycle). All aquaria had a gravel substrate and were aerated via an air stone and tubing connected to an air pump. The aquaria had a dark coloured, rectangular, plastic shelter tube with one long side cut out for viewing (12×12×12 cm) placed on the bottom in the rear centre of the aquaria. All aquaria had yellow tape placed along the outer edges of the tank. The tape had black lines drawn on with 1 cm distances between each line. This provided a method to estimate swimming distance when analysing behaviours.To visually isolate the cod from one another and from other disturbance, opaque black panels on the sides separated the aquaria and dark fabric covered the front. Each fish was acclimated for seven days in its aquarium before the treatment trial commenced. On the seventh day treatment trials were commenced at the same time each day with one fish at 8∶00 am and a second fish at 10∶30am. A total of six treatment groups were used with six to eight cod in each group; control treatment 1 (Saline, N = 7), treatment 2 (0.1% Acetic acid, N = 7), treatment 3 (2.0% Acetic acid, N = 7), treatment 4 (0.005% Capsaicin, N = 6), treatment 5 (0.1% Capsaicin, N = 8) and treatment 6 (Fishing hook, N = 7). The chosen chemical stimuli and their concentrations were based on pilot studies and on existing literature on nociception and potential pain in other teleost fish species (*e.g.* acetic acid: [15], [20], [22]–[24]) and in studies of nociception and potential pain in human pain research (*e.g.* capsaicin: see [21], [32]–[34]). All treatments were applied to the lip of the fish, as this area has also been used in similar studies in other fish species (*i.e.* [15], [16], [20], [22]–[24], [35]) and nociceptors were identified on the lips of the rainbow trout [13]–[15]. Each fish underwent one treatment only and all treatments were randomly assigned. The procedure consisted of the single cod being quickly but carefully netted from its aquarium and into a 5 L anaesthetic bath (40 mg/L; metacaine (MS-222), Pharmaq AS, Oslo, Norway). Metacaine [36] was chosen as it has been used in comparable previous studies with freshwater fish, is comparable to other drugs used [14]–[16], [20] and has well-documented effects in the Atlantic cod including rapid induction and recovery times [37]. When deep plane anaesthesia was attained i.e. loss of tail reflex and lack of response to tail pinch, the designated treatment was applied. After treament the cod was returned to its aquarium. The time lapse from dip-netting to being returned to the aquarium was always less than 180 sec, and the fish regained equilibrium within 529±43 sec after reimmersion. For the fish assigned to treatment groups 1 to 5, 25 µl of the chemical solution was injected subcutaneously to the upper lip, and 25 µl was injected to the lower lip, always on the left side, 10 mm from the front center of the lips, using a sterile 250 uL N Syringe with cemented needle (725N 250 µl SYR (22s/2″/2), Hamilton Bonaduz AG, Switzerland). The needle was inserted into the subcutaneous connective tissue of the lip before the injection took place. For the fish assigned to treatment group 6, a standard recreational saltwater fishing hook (40 mm from eye to bend, 10 mm gape, straight hook eye w/cutting point, Mutu Circle Hooks, Australia) was inserted from the mouth opening side going through the thin elastic fold of the skin and then through the lower left subcutaneous tissue of the lip (*i.e.* in the same position as the injection treatments), and remained attached to the cod for the entire experiment. Following the 120 min post-treatment observation period, the cod was quickly dip-netted and killed by an overdose of metacaine (160 mg.L**^−^** ^1^), and a blood sample was taken. Time between cod being dipnetted and killed by metacaine was 180±30 sec. Finally, the fish was weighed (Top Pan Precision Balances, PGL 4001, Adam Equipment, Danbury CT, USA), standard length measured to 0.1 cm, and the site of injection/hook inspected to notice possible physical effects of the treatment.

### Measurements of Opercular Beat Rate (OBR)

Twenty minutes (−20 to −14 ) before treatment and at at the time points 10 min (10–16), 30 min (30–36), 60 min (60–66), 90 min (90–96), and 120 (114–120) min after treatment, OBR was measured by counting the number of opercular beats per minute during three intervals (one minute between each interval) so an average OBR could be calculated for that observation period. To ensure the cod were not disturbed, OBR was observed from a TV-screen in an adjacent room by means of a digital video camera placed in front of the aquarium (described below).

### Behavioural Observations

Digital wide-angle lens video cameras (Sony Handycam, DCR-SR47 HDD, Toyko, Japan) were placed in front of the glass aquaria for counting opercular beat rates and recording behaviour. The cameras were placed in front of tanks 18 hours prior to commencement of each trial to allow habituation. Each fish was continuously recorded from 30 min pre-treatment until 120 min post-treatment. From these video recordings, behavioural data were either measured by the percentage of time the fish engaged in a behaviour or by the number of times a behaviour was observed according to a simple ethogram (Table 1). The ethogram contains the most common types of behaviours observed during pre and post treatments. The behaviours were scored for the 15 min period prior to treatment (10–25 min) and from (30–45 min) and (90–105) min after the treatment. High reliability and validity of these measurements was tested and demonstrated by intra- observer reliability tests preformed on two of the videos (intra- r^2^ = 0.859, r^2^ = 0.998; Spearman Rank correlation) and inter-observer reliability tests performed on three of the videos chosen at random (inter- r^2^ = 1.000; r^2^ = 0.967, r^2^ = 0.994; Spearman Rank correlation).

**Table 1:** Ethogram presenting the categorization of behaviors in the present study. Behavioural data are expressed as the percentage duration (%) or counts of episodes (#).

Category	Measurement	Description
Shelter	Duration (%)	Positioned with 75% of body under shelter located on bottom of aquaria
Hovering top	Duration (%)	Hovering and/or stationary in top 15% portion of water column
Hovering bottom	Duration (%)	Hovering and/or stationary on bottom 15% portion of water column
Swimming	Duration (%)	Swimming around entire tank, moving more than one body length per second
Head shaking	Count (#)	Episodes with a series of very rapid lateral movements of the head.

### Measurement of Blood Parameters

A sample of blood (1.0–1.5 ml) was taken from the caudal vasculature using a heparinised sterile 2.0 ml syringe (BD Plastipak, UK) and 21G**×**1 ½ ˝ hypodermic-needle (Braun, Switzerland). Whole blood was immediately analysed using hand-held meters evaluated for use in fish [38], [49], and frequently used in studies in the Atlantic cod (*e.g.* [40], [41]). Lactate levels were measured using Lactate Pro™ (Arkray, Kyoto, Japan) whereas hematocrit and concentrations of sodium (Na+), potassium (K+) and glucose were measured using an i-STAT Portable Clinical Analyzer (Abbot Point of Care Inc.; Princeton, NJ, USA). The remaining blood was placed on ice in a 4.0 ml lithium-heparinized vacuum tube (BD Vacutainer, Franklin Lakes, NJ, USA), centrifuged (8 min, 2700×g at 4.0°C, Beckman Coulter Avanti J-20XP Centrifuge, USA) and plasma frozen and stored at −20°C for later analysis of cortisol levels. Plasma cortisol was measured by means of radioimmunoassay (RIA) according to a protocol recently validated for Atlantic cod (Dr. H. Tveiten, personal communication Nofima AS, Muninbakken 9–13, NO-9291, Tromsø, Norway). Briefly, cortisol was extracted from 200 µl of plasma with 4 ml diethyl ether under vigorous shaking for 4 min. The aqueous phase was frozen in liquid nitrogen, whereas the organic phase was transferred to a glass tube, evaporated in a water bath at 45°C and then reconstituted by addition of 600 µl assay buffer and then assayed according to established protocols [42], [43]. The detection limit of the assay was 0.6 ng·ml**^−^** ^1^.

### Chemicals

Solutions were dissolved in saline (Ringers solution). The acetic acid solutions were made on the day of the experiments to ensure the solutions were not neutralized by the saline. The 2% acetic acid was made by diluting 200 µl of glacial acetic acid (laboratory grade, VWR International, Oslo, Norway) in 10 ml of Ringer’s solution (pH = 2.5). The 0.1% acetic acid was made by diluting 10 µl of glacial acetic acid in 10 ml of Ringer’s solution (pH = 3.2). Capsaicin was obtained from Sigma-Aldrich Norway AS (Oslo, Norway). To ensure solubility of capsaicin, the capsaicin solutions were prepared in one dimethyl sulfoxide (DMSO) (D5879-Sigma Aldrich Co, St. Louis, MO, USA,) to give a final concentration of 1% DMSO in Ringers. A stock solution of capsaicin was prepared by dissolving 10 mg capsaicin in 0.1 ml DMSO. The 0.1% capsaicin treatment solution was made by adding 10 µl of capsaicin stock solution in 100 µl of DMSO and diluting in Ringer’s solution to give a total volume of 10 ml. The 0.005% capsaicin was made by adding 50 µl of the 0.1% capsaicin/Ringers solution in 105.5 µl of DMSO and diluting in Ringer’s solution to give a total volume of 10 ml. Ringer’s physiological solution was made by mixing distilled water with (g·L**^−^** ^1^) NaCl 12.85, KCL 0.29, MgCl_2_ 0.20, CaCl_2_ 0.29, and Hepes 2.38; pH was adjusted to 7.2. All the chosen acetic acid and capsaicin concentrations used were tested on a limited number of cod in a preceding pilot study to ensure sufficent effectiveness. It was found that injection of 25 µl of the high concentrations of both acetic acid and capsaicin into the lip of Atlantic cod caused white spots which progressed into necrosis within a few days. Accordingly, it was concluded from these tests that the high concentrations of these chemicals were sufficiently high to act as noxious stimuli and that higher concentrations were not needed.

### Data and Statistical Analysis

Statistical analyses (p≤0.05 considered statistically significant) were performed using SPSS version 19 software (IBM Corporation, Armonk, NY, USA). All data were tested for normal distribution using Kolmogorov-Smirnov test and homogenity of variances was tested using Levene’s *F* test and those that met parametric assumptions were tested accordingly whereas those that did not were analysed using non-parametric statistics. To determine if there were any differences in the physiological parameters between the fish tested at 8∶00 am and the fish tested at 10∶30 am independent t-tests were conducted (p≤0.05). OBR data were tested for differences between treatment groups at each time-point (*i.e.* at 20 min prior to treatment application and at 10, 30, 60, 90 and 120 min following treatment application) using two-way repeated measure ANOVA with a Greenhouse-Geisser correction followed by post-hoc tests with a Bonferroni correction. Greenhouse-Geisser correction was used as it is conservative in incorrectly rejecting the null hypothesis. To determine differences in OBR between the before and after effects of each treatment, a repeated measures ANOVA was applied again using a Greenhouse-Geisser correction followed by post-hoc tests using the Bonferroni correction. Video recordings were analyzed for behaviours using Adobe Premiere Pro CS4.1 (Adobe Systems Incorporated, USA). Possible differences between and within treatment groups in the occurrence of the categorized behaviours (Table 1) were tested using a repeated measures general linear model (GLM) followed by post-hoc tests using the Bonferroni correction. Blood parameters were tested to determine any differences between treatment groups using one-way ANOVA for normally distributed data and Kruskal Wallis tests for non-normally distributed data.

## Results

### Opercular Beat Rate (OBR)

**Figure 1:**
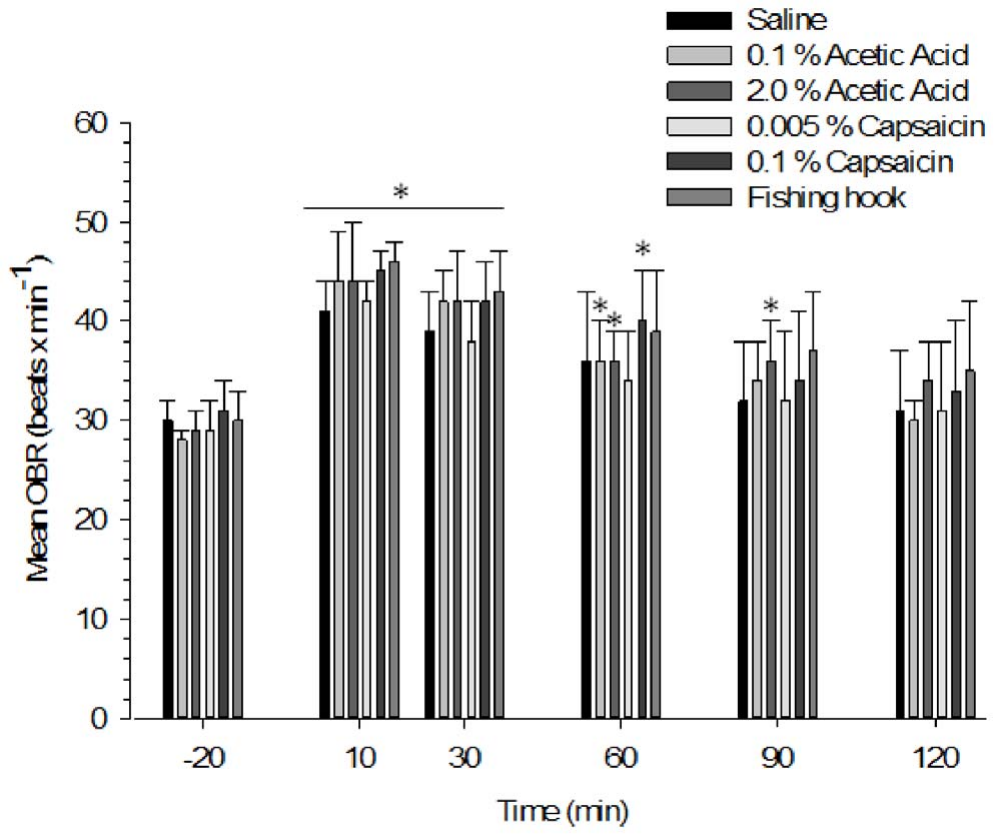
Opercular beat rates (beats×min^−1^; mean OBR±S.E.) for each treatment group at 20 min pre-treatment (−20 min) and at 10, 30, 60, 90 and 120 min post-treatment (i.e. corresponding to the data presented in Table S1). There were no statistically significant differences (p>0.05) in OBR evidenced between treatment groups at any time-point (two-way repeated measures ANOVA with a Greenhouse-Geisser correction followed by post-hoc test using the Bonferroni correction). Asterisks (*) denote a statistically significant (p≤0.05) within-group difference in OBR compared to its respective pre-treatment recording (one-way repeated measures ANOVA with a Greenhouse-Geisser correction followed by post-hoc test using the Bonferroni correction). N = 7 fish per group except for 0.005% Capsaicin (N = 6) and 0.1% Capsaicin (N = 8).

OBR in the cod increased after treatment in all groups (Figure 1; Saline (F(3, 16) = 28.721, p<0.001), 0.1% Acetic Acid (F(2, 13) = 43.434, p<0.001), 2.0% Acetic Acid (F(2, 15) = 29.882, p<0.001), 0.005% Capsaicin (F(2, 9) = 12.398, p<0.001). 0.1% Capsaicin (F(2, 14) = 27.062, p<0.001), Hook in lower lip (F(3, 16) = 19.994, p<0.001). Post hoc tests with the Bonferroni correction showed that from 10 min post treatment onwards, the OBR steadily declined with time and recovered to approximately normal (p>0.05 difference from its pre-treatment level) between 60 min (Saline, 0.005% Capsaicin and Hook in lower lip), 90 min (0.1% Acetic acid and 0.1% Capsaicin) and 120 min (2% Acetic acid) post treatment (Figure 1; Table S1). At any given time point, there were no statistically significant (p>0.05) differences in OBR between the saline treatment group (control) and any of the other treatment groups (Figure 1; Table S1).

### Behavioural Observations

**Figure 2:**
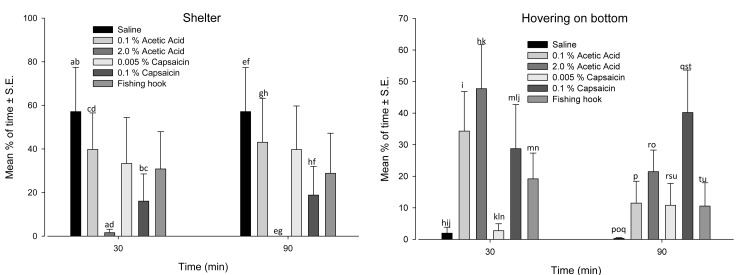
Occurrence of Shelter and Hovering on the bottom behaviours in Atlantic cod before and after saline, acetic acid, capsaicin, and fishing hook treatments. The data are expressed as mean percentage of time (%,**±**S.E.) the behaviour was displayed during 15 min segments at 30 min and 90 min after treatment. For each time point, identical letters denote a statistically significant (p≤0.05) difference between treatment groups (repeated measures GLM followed by post-hoc test using the Bonferroni correction). N = 7 fish per group except for 0.005% Capsaicin (N = 6) and 0.1% Capsaicin (N = 8).

During the pre-treatment period all of the categorized behaviours (Table 1) were observed except for head-shaking. Two types of behaviors were significantly affected by treatments: hovering on the bottom of the aquaria (F(5,108), = 4.709 p = 0.001) and use of shelter (F(5,108) = 2.427, p = 0.050); (Figure 2; Table S2). Post hoc tests (mean % of time**±**S.E.) for use of shelter using the Bonferroni correction showed that 2.0% Acetic acid significantly reduced percentage of time the sheltering behaviour was observed (1.6% ±1.6 at 30 min and 0.0±0.0% at 90 min) compared with 0.1% Acetic acid (39.8±16.7% at 30 min and 43.1±20.1% at 90 min) (p = 0.033) and Saline (57.1±20.2% at 30 and 90 min) (p = 0.021). 0.1% Capsaicin also decreased the % of time the sheltering behaviour was observed (16.0±12.5% at 30 min and 18.8±13.1% at 90 min) compared with 0.1% Acetic acid (39.8±16.7% at 30 min and 43.1±20.1% at 90 min) (p = 0.020) and Saline (57.1±20.2% at 30 and 90 min) (p = 0.013). Post hoc results (Bonferroni correction, mean % of time**±**S.E.) associated with hovering on the bottom demonstrated that 2.0% Acetic Acid significantly increased the percentage of time (47.8±14.0% at 30 min and 21.5±6.8% at 90 min) that cod were observed hovering on the bottom of the aquaria as compared with 0.005% Capsaicin (2.8 2.2% at 30 min and 10.8±7.0% at 90 min) (p = 0.010) and Saline (2.0±1.9% at 30 min and 0.3±0.2% at 90 min) (p = 0.001). Further 0.1% Capsaicin increased the percentage of time (28.7±14.0% at 30 min and 40.2±13.5% at 90 min) cod were observed hovering on the bottom of the aquaria as compared with 0.005% Capsaicin (2.8±2.2% at 30 min and 10.8±7.0% at 90 min) (p = 0.02), Hook (19.2±8.0% at 30 min and 10.6±7.3% at 90 min) (p = 0.021) and Saline (2.0±1.9% at 30 min and 0.3±0.2% at 90 min) (p<0.001). Also, 0.1% Acetic acid increased the mean percentage of time (34.3±12.6% at 30 min and 11.5±6.7% at 90 min) cod were observed hovering on the bottom of the aquaria compared with Saline (2.0±1.9% at 30 min and 0.3±0.2% at 90 min) (p = 0.032). The analysis demonstrated that only the behaviour of hovering in the top of the tank was affected by time (F (2, 108)_ = _3.092, p = 0.050) with post hoc analysis showing that the total mean percentage of time this behavior was observed was significantly different between the pre-treatment observations (6.69±2.69 (mean % of time**±**S.E.)) and those at post 90 min (20.14±4.74 (mean % of time**±**S.E.); p = 0.017) for all treatments. There was no interaction of time and treatment on any of the behaviors.

Only cod that had a hook inserted into the lower lip displayed episodes of head-shaking. Each episode lasted 1 - 2 seconds and consisted of a few very quick sideway movements of the head (the movements were too quick to be counted based on the video recordings). Head shaking episodes were not observed in any fish pre-treatment, but a total of 12 episodes (range 0–6 episodes per fish) were observed in five of the seven fish during the 30–45 min observation segment and again a total of 10 episodes (range 0–4 episodes per fish) in four of the same five fish during the 90–105 min observation segment. In all fish, the hook remained in place for the entire length of the experiment.

### Blood Parameters

There were no statistically significant (p≤0.050) differences in physiological blood parameters (plasma cortisol and whole blood lactate, glucose, sodium, potassium and hematocrit) between treatment groups at termination of the experiment at 120 min post-treatment (Table 2).

**Table 2:** Whole blood parameters and plasma cortisol levels in Atlantic cod at 120 min post-treatment.

Treatment	Saline	0.1% Acetic acid	2.0% Acetic acid	0.005% Capsaicin	0.1% Capsaicin	Fishing hook
*Cortisol (ng·ml^−1^)	54.4±32.7	31.9±22.2	59.3±27.8	12.2±18.4	31.0±24.0	44.0±32.7
*Lactate (mmol·L^−1^)	1.9±1.9	1.8±0.8	2.7±1.4	1.5±0.6	1.7±0.6	1.7±0.6
**Glucose (mmol·L^−1^)**	3.7±0.7	3.5±0.8	3.8±1.6	3.4±1.4	3.3±0.8	3.4±0.8
**Na^+^ (mmol·L^−1^)**	160.9±4.5	155.1±10.0	159.9±2.4	158.5±4.0	158.8±9.0	159.0±4.0
*K^+^ (mmol·L^−1^)	3.6±0.6	3.5±0.6	3.7±0.6	3.2±0.6	3.6±0.7	3.4±0.4
**Hematocrit (%)**	20.0±2.5	17.9±3.4	21.9±2.5	19.0±3.4	17.8±3.5	18.6±3.0

Data are expressed as mean (±S.E.) for normally distributed data and as medians (±IQR) for non-normally (*) distributed data. There were no statistically significant differences between treatment groups for both normally distributed data (one-way ANOVA) and not normally distributed data (Kruskal Wallis). N = 7 fish per group except for 0.005% Capsaicin (N = 6) and 0.1% Capsaicin (N = 8).

## Discussion

### Responses to Acetic Acid

Reduced pH of the extracellular fluid is associated with pathophysiological conditions such as inflammation, hypoxia and anoxia, and cutaneous injection of low pH solution causes prolonged activation of sensory afferents which may cause a sharp stinging pain in humans [44]. In fish, acetic acid is found to activate sensory nociceptive afferents [16] and this stimulus has been used in studies of rainbow trout (*O. mykiss*), zebrafish (*D. rerio*), common carp (*C. carpio*) and goldfish (*C. aurata*). In the present study, injection of 2% acid into the Atlantic cods lips reduced the amount of time spent under shelter and both 0.1% and 2% acetic acid increased the amount of time spent hovering near the bottom of the aquaria, as compared to saline-injected control. There were, however, no differences in circulatory stress indicators or opercular beat rate (OBR) at any time-point between saline-treated controls and acid-injected fish.

Although all treatments including saline control resulted in temporarily increased OBR, the injection of acetic acid was associated with a slight delay in this recovery to pre-treatment OBR-values with fish given 0.1% acid recovering in 90 minutes and fish given 2% acid taking in 120 minutes to recover, compared to 60 min in the saline control group.

In rainbow trout [5], [19], [22] and zebrafish [19] the injection of acetic acid into the lip resulted in an opercular beat rate (OBR) that was greater than saline controls and took significantly longer to return to normal values. In the cyprinids goldfish and carp, however, OBR was found not to be elevated above controls following the injection of acetic acid, even when much higher doses (5 and 10%) of acetic acid were used in the carp [17], [20]. Possibly, the lack of differences between control and noxiously stimulated Atlantic cod in OBR and circulatory stress parameters may be linked to consistent individual variation known to exist in both behaviour and stress physiology in a variety of species including fish [45]–[47]. Such differences have been recorded during acid injection in rainbow trout after noxious treatment [35] but has not been investigated in cod. Similarly, the effects of acid injection on the use of shelter has been investigated in rainbow trout and carp but with inconsistent results [14], [20], [23].

Interestingly, our cod did not display any changes in rapid swimming behaviour in the present study. This is in contrast to a prolonged decrease in swimming activity with acid injection in zebrafish and rainbow trout [20] and an immediate and vigorous increase in swimming activity in unanaesthetized rainbow trout and goldfish [17], [22], but corresponds to unaffected swimming in the common carp [20]. In line with this, the lack of differences in circulatory stress parameters in the present study corresponds to previous laboratory studies which have found that unstressed cod could be described as hovering or swimming slowly in the water column whereas stressed fish displayed fast swimming, jumping or laying still on the bottom [48]–[50].

In common carp, rainbow trout, and goldfish, the injection of acetic acid was associated with observations of anomalous behaviours such as sideways rocking body movements and/or rubbing of the affected areas against the tank wall or in the gravel [17], [19], [20]. No such behaviours were observed in Atlantic cod in this study or in zebrafish after injection of 5% acetic acid into the lips [22]. One cannot exclude, however, that this may reflect a species-specific difference with anomalous behaviours being avoided as they may attract the attention of predators [20], [51], and future studies could explore if anti-predator behaviour is disrupted in Atlantic cod during a noxious stimulation.

Responses to higher concentrations of noxious chemicals often elicit a greater response from the animals. In line with this, the more prolonged recovery of OBR, the reduced use of shelter and the increased time spent hovering in the bottom of the aquaria seen in cod given 2% acid compared to 0.1% acid may be expected. In our cod, the acetic acid as well as the capsaicin concentrations used were tested in a preceding pilot study where it was found that the high concentrations of these chemicals (but not the chosen low doses) caused white spots which progressed rapidly into necrosis, indicating that the high concentrations used were sufficiently high to act as noxious stimuli. In contrast, investigations using common carp and zebrafish have used much higher doses of acetic (5–10%) to elicit a behavioural response. Comparably, injections of 0.7% acetic acid in the cheek of goldfish was reported to cause tissue damage with the injection site turning white, and was associated with an immediate vigorous escape and rubbing behaviour which was reduced with the administration of morphine [17]. We cannot exclude the possibility, however, that higher concentrations may have elicited more pronounced responses in cod.

Although some physiological parameters were unaffected possibly due to time of sampling, the delayed recovery of OBR, the reduced use of shelter and the increased hovering in the bottom of the aquaria differentially induced by 0.1% and 2% acetic acid in our cod correspond to responses seen with acetic acid in rainbow trout, zebrafish and goldfish, possibly representing responses specific to the detection of injurous stimuli.

### Responses to Capsaicin

Capsaicin was included in the present study because it is a known potent agonist for the vanilloid TRPV1 receptor (previously called VR1) and may produce dose-dependent long-lasting intense burning pain in humans [32], [33], [52], [53]. Recently, the presence of vanilloid-family receptors has been demonstrated in goldfish [54], zebrafish [54], [55] and the European sea bass, (*Dicentrarchus labrax*) [56] but capsaicin has not previously been explored as a nociceptive stimulus in fish. Of the two doses of capsaicin tested in the present study, only Atlantic cod which received the higher dose (0.1%) displayed significant effects, consisting of a reduced use of shelter, increased hovering near the bottom of the aquaria, and an indicated slight delay in the recovery of OBR to pre-treatment levels (90 minutes with 0.1% capsaicin compared to 60 minutes for 0.005% capsaicin and saline control). This demonstrates that 0.1% capsaicin is able to stimulate sensory afferents in the lip of the Atlantic cod. Although responsiveness to capsaicin may suggest the presence of TRPV1 receptors and a possible stimulation via C-fibre nociceptors [34], [57] this was not subject to investigation in the present study and therefore remains unknown. The presence of C-fibres has been documented in fish [13], [19] and similar histological and electrophysiological investigations are underway also in the Atlantic cod (Bichão *et al*., unpublished data). The responses to capsaicin in our cod generally were mild but the responses observed with 0.1% capsaicin corresponded to that seen with acetic acid and may support the assumption of being specific to noxious stimulation.

### Responses to a Fishing Hook

The insertion of a fishing hook through the lip of a fish undoubtedly causes tissue damage and should be regarded a noxious stimulus. In humans, punctate mechanical stimuli may cause the experience of sharp pain [58], [59]. In the present study, the only effect observed in hooked Atlantic cod was episodes of head shaking movements which were not seen pre-treatment or in the other treatment groups. This demonstrates that the presence of the hook is detected by the cod and induces a behavioural change, possibly seeking to get rid of it. The lack of other physiological and behavioural effects to the inserted hook suggests that the mechanical tissue damage *per se* may contribute little to reported angling-induced responses in this species, and that other factors such as pull force, physical exhaustion and stress responses to play, landing, air exposure and subsequent handling may be more influential on the physiology and behviour of angled fish [7], [60], [61].

In a previous laboratory study by Fernö and Huse [62], head-shaking episodes similar to those described in the present study were reported during repeated capture of wild-caught cod by a baited hook. In that study, the proportion of cod taking the hook was reduced with successive trials and it was concluded that aversive physical stimulation from the hook was the main negative reinforcement in this conditioning [63]. However, although Atlantic cod are found to display impressive associative learning capability and long time memory retention [64], the study by Fernö and Huse [62] found that individual Atlantic cod repeatedly encountered a baited hook over several days in two sets of experiments separated by almost 6 months [62], demonstrating a low hook avoidance in Atlantic cod compared to reports from some other freshwater species (e.g. [65], [66]). In line with this, the absence of observable responses other than head shaking to the presence of the hook in the present study may suggest a resiliency to hook damage in the Atlantic cod. Indeed, the almost complete absence of observable responses to punctate mechanical injury of the lip in the present study may be related to the eating habits of cod, which include species with hard or pointed components such as mussels, clams, whelks, brittle stars and crustaceans [67]. Notably, these observtions with puncture of the lip of the Atlantic cod may also correspond to a similar observation during puncture of the skin of the head of White Sea cod (*Gadus ogac*), that did not cause as strong responses when compared with stimulation of the fins and olfactory epithelium [68].

### Use of Anaesthesia

In the present study, as in most previous studies on responses to noxious stimuli in fish (*e.g.* [15], [17], [20], [22], [53], [69]) noxious treatments were applied under acute anaesthesia so that the treatments could be applied to a precise area and to facilitate humane handling of the animals. However, the use of anaesthesia will also provide temporary analgesia, thus masking acute effects of the tissue damage and possibly impeding longer term responses [16], [36]. In a study using un-anaesthetized rainbow trout, the injection of acetic acid into the lip caused an immediate and temporary loss of equilibrium in two of eight fish at a concentration of 2% and in seven of eight fish at a concentration of 5% [22]. Apart from this, effects on swimming behaviour and changes in OBR for the unanaesthetized rainbow trout were similar to those reported for anaesthetized rainbow trout [15]. This demonstrates that chemical stimuli may have the potential to remain in the tissue and continue stimulating sensory afferents well beyond the time of analgesic effects induced by the anaesthetic [23], [70]. The introduction of the fishing hook may have a similar effect, implying that any observed responses are not to the initial puncture of the tissue per se, but rather to the persisting mechanical damage and possible stimulations by movements of the hook in the awake fish. Although we cannot exclude the possibility that the anaesthetic used may have delayed responses more than 2 hours post-treatment, it has been demonstrated that the anaesthetic used did not influence commonly used behavioural parameters and that it may be possible to start behavioural observations even sooner than 30 min after anaesthesia [25].

### Impact on Physiological Stress

Although the use of anaesthetic served to minimize stress and ensure rapid and standardised stimulus administration, the confinement, handling, anaesthesia and asphyxia may still initiate a stress response irrespective of treatment group [36], [71], [72]. In line with this, OBR is typically elevated with stress [28], [48], and a temporary post-treatment increase in this parameter was observed in all treatment groups in the present study. Surprisingly, no significant differences were found in the levels of circulatory stress indicators in our Atlantic cod two hours post-treatment. This suggests that none of the applied noxious stimuli may be considered significantly more stressful than the handling and administration of the innocuous control stimulus. Similarly, a general lack of differences in stress indicators between treatment groups was reported for Nile tilapia (*O. niloticus*) where OBR and circulatory stress parameters did not differ between tailfin-clipped fish and controls that were only handled [19]. For noxiously stimulated rainbow trout only acid injected fish that were held individually exhibited an elevation in plasma cortisol levels compared with controls. That was not observed in fish held in social groups although cortisol values were much higher in group housed fish [16], [37]. Because these studies all use terminal sampling at the end of the experiments and in the case of trout when all behavioural and physiological effects had subsided 3 hours after treatment, it is possible that the fish had recovered or adapted to the treatment.

### Conclusions

This study has found that acetic acid, capsaicin and the presence of a fishing hook in the lip of Atlantic cod did affect behaviour when compared to the responses seen with the innocuous control treatment. Only head shaking was seen in the hook insertion group and whether this reflects a real resiliency to tissue damage in the mouth area remains to be tested. Given the diet of cod a lack of response to the hook may be related to the eating habits of this species. Still, the delayed recovery of OBR, the reduced use of shelter and the increased hovering behaviour induced by both acetic acid and capsaicin correspond to previous observations with the injection of acetic acid in rainbow trout, zebrafish and goldfish possibly representing indicators to assess injury detection and potentially aversive events in Atlantic cod. The lack of all these responses apart from head-shaking events in hooked fish possibly indicate that the fish are aware of the presence of the hook, and possibly seek to remove it from their lips. Future studies should explore noxious stimulation in the context of individual differences in stress coping styles and in a more realistic angling context and show the presence of nociceptors.

## Supporting Information

Table S1 **Pre-treatment OBR (beats×min^−1^; mean±S.E.M.), mean increase in post-treatment OBR (±S.E.M.), and the statistical significance (P-value) for the within-group comparison of post-treatment OBR with the corresponding pre-treatment value at each time point.** Recovery time represents the time-point at which post-treatment OBR no longer differed significantly (p>0.05) from the pre-treatment measures (one-way repeated measures ANOVA with a Greenhouse-Geisser correction followed by post-hoc test using the Bonferroni correction). There were no statistically significant differences (p>0.05) in OBR evidenced between treatment groups at any time-point (two-way repeated -Geisser correction followed by post-hoc test using the Bonferroni correction). N = 7 fish per group except for 0.005% Capsaicin (N = 6) and 0.1% Capsaicin (N = 8).
(TIF)

Table S2 **Occurrence of Shelter and Hovering on the bottom behaviours in Atlantic cod before and after saline, acetic acid, capsaicin, and fishing hook treatments (i.e. corresponding to the data presented in** **Fig. 2** **).** The data are expressed as mean percentage of time (%,**±**S.E.) the behaviour was displayed during 15 min segments prior to treatment administration (−20 min) and at 30 and 90 min after treatment administration. For each behaviour, identical letters denote a statistically significant (p≤0.05) difference between treatment groups at the same time point (repeated measures GLM followed by post-hoc test using the Bonferroni correction). N = 7 fish per group except for 0.005% Capsaicin (N = 6) and 0.1% Capsaicin (N = 8).
(TIF)
