# Supplementary figures and images for: Physiological and Behavioural Responses to Noxious Stimuli in the Atlantic Cod (Gadus morhua)
Source: PLoS One. 2014 Jun 17;9(6):e100150. doi: 10.1371/journal.pone.0100150 (PMC4061104; doi:10.1371/journal.pone.0100150)

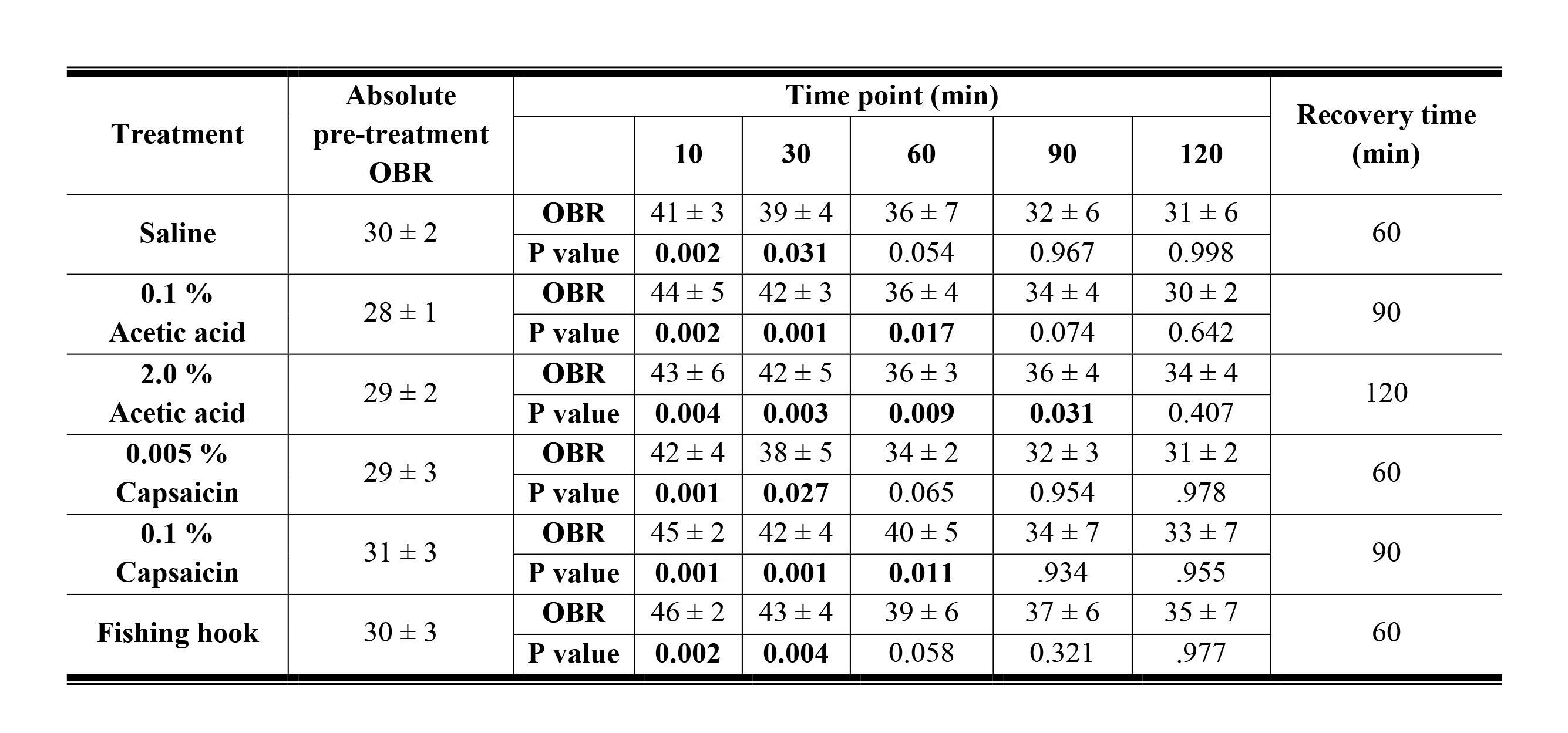

Supplement: Table S1 — Pre-treatment OBR (beats×min−1; mean±S.E.M.), mean increase in post-treatment OBR (±S.E.M.), and the statistical significance (P-value) for the within-group comparison of post-treatment OBR with the corresponding pre-treatment value at each time point. Recovery time represents the time-point at which post-treatment OBR no longer differed significantly (p>0.05) from the pre-treatment measures (one-way repeated measures ANOVA with a Greenhouse-Geisser correction followed by post-hoc test using the Bonferroni correction). There were no statistically significant differences (p>0.05) in OBR evidenced between treatment groups at any time-point (two-way repeated -Geisser correction followed by post-hoc test using the Bonferroni correction). N = 7 fish per group except for 0.005% Capsaicin (N = 6) and 0.1% Capsaicin (N = 8). (TIF) [file pone.0100150.s001.tif]

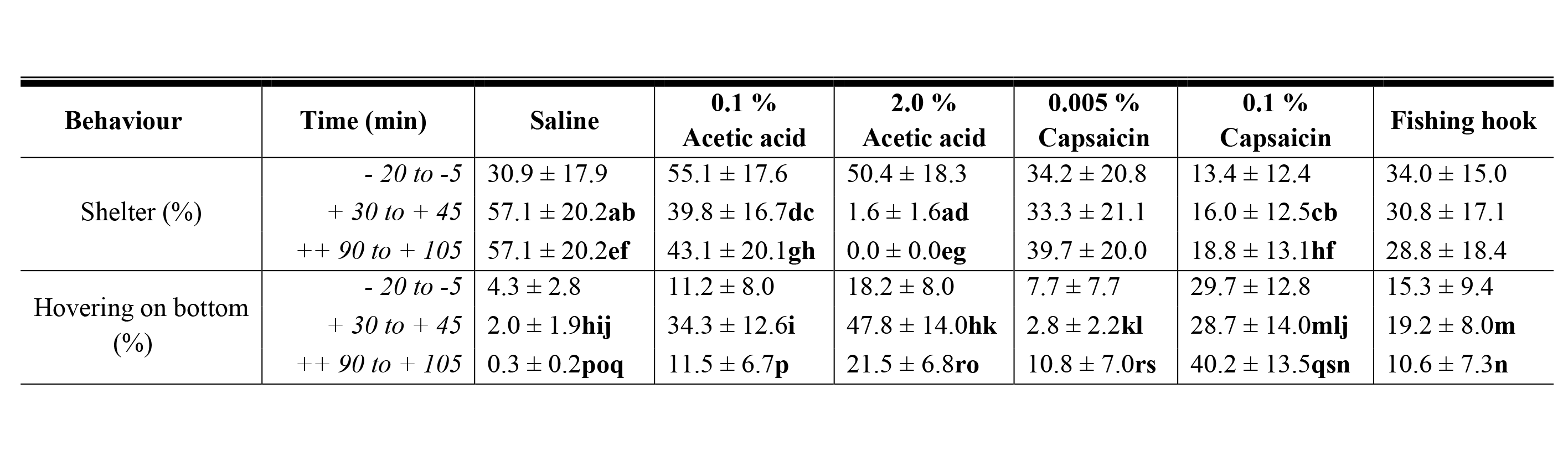

Supplement: Table S2 — Occurrence of Shelter and Hovering on the bottom behaviours in Atlantic cod before and after saline, acetic acid, capsaicin, and fishing hook treatments (i.e. corresponding to the data presented in Fig. 2 ). The data are expressed as mean percentage of time (%,±S.E.) the behaviour was displayed during 15 min segments prior to treatment administration (−20 min) and at 30 and 90 min after treatment administration. For each behaviour, identical letters denote a statistically significant (p≤0.05) difference between treatment groups at the same time point (repeated measures GLM followed by post-hoc test using the Bonferroni correction). N = 7 fish per group except for 0.005% Capsaicin (N = 6) and 0.1% Capsaicin (N = 8). (TIF) [file pone.0100150.s002.tif]
